# Genome-wide identification and expression profile of *CYP* genes in rubber tree (*Hevea brasiliensis*)

**DOI:** 10.3389/fpls.2026.1750005

**Published:** 2026-04-22

**Authors:** Qianyi Lyu, Zhi Deng, Tingkai Wu, Yuan Yuan, Wanqi Liang, Han Cheng

**Affiliations:** 1 Yazhou Bay Institute of Deepsea Science and Technology, Shanghai Jiao Tong University, Sanya, China; 2 National Key Laboratory for Tropical Crop Breeding, Ministry of Agriculture and Rural Affairs Key Laboratory of Biology and Genetic Resources of Rubber Tree, Rubber Research Institute, Chinese Academy of Tropical Agricultural Sciences, Haikou, China; 3 Sanya Research Institute, Chinese Academy of Tropical Agricultural Sciences, Sanya, China; 4 State Key Laboratory Incubation Base for Cultivation and Physiology of Tropical Crops, Rubber Research Institute, Haikou, China

**Keywords:** CYP, genome evolution, *Hevea brasiliensis*, meJA and ethylene, rubber biosynthesis

## Abstract

**Introduction:**

Cytochrome P450 monooxygenases (CYPs) are crucial in plant secondary metabolism, catalyzing diverse biochemical reactions, defense, and stress adaptation. Hevea brasiliensis is the primary source of natural rubber, but its CYP family remains underexplored, which may play essential roles in rubber biosynthesis and environmental tolerance.

**Method and Result:**

n this study, we systematically identified 238 HbCYP genes in H. brasiliensis, classified them into 9 clans and 43 subfamilies, with Clan 71 contraction and Clan 72 and Clan 85 emerging as the most expansive clans. Gene structure analysis revealed that 68.87% of A-type HbCYPs have one intron. Separately, analysis of conserved motifs in promoter regions identified a high abundance of cis-acting elements, including 456 related to methyl jasmonate (meJA) responsiveness and 3,302 related to light responsiveness. Genome evolution analysis indicated that 64 tandem and 119 WGD or segmental duplications significantly contributed to the expansion of the HbCYP family, also supported by orthologous genes with other four species. Tissue-specific profiling revealed differential HbCYPs expression across H. brasiliensis organs. Notably, meJA and ethylene (ET) treatments differentially expressed 93 and 60 HbCYP genes, respectively. Gene Ontology (GO) analysis and real-time quantitative polymerase chain reaction (RT-qPCR) validation of these genes revealed their significant contribution in rubber biosynthetic pathways. Protein interaction networks highlighted collaborations between HbCYPs and key rubber biosynthesis enzymes, including 3-hydroxy-3-methylglutaryl-CoA synthase (HMGS), and these results demonstrate functional diversification within the HbCYP family associated with rubber biosynthesis.

**Conclusion:**

Collectively, this study provides the first comprehensive genome-wide analysis of the HbCYP family in H. brasiliensis, offering insights into their evolutionary dynamics and functional diversification. The results establish a foundation for future research on CYP-mediated rubber biosynthesis and stress adaptation, with implications for molecular breeding of high-yield and stress-tolerant rubber tree cultivars.

## 1 Introduction

The CYP superfamily constitutes one of the largest enzyme families in nature, first identified through their distinct spectral absorption at 450 nm upon binding carbon monoxide in rat liver microsomes (Klingenberg, 1958; Omura and Sato, 1962). These heme-containing monooxygenases catalyze oxidation reactions, hormone biosynthesis, and secondary metabolism, which play critical roles in the growth, development, and stress response of organisms at various life stages (Chakraborty et al., 2023). Their structural plasticity, characterized by conserved catalytic cores and variable substrate-binding regions, allows them to process a vast array of substrates, including lipids, steroids, and xenobiotics (Werck-Reichhart and Feyereisen, 2000). A comprehensive collection of over 300,000 CYP protein sequences has been compiled from diverse sources, including viruses, aquatic organisms, plants, fungi, bacteria, and archaea (Nelson, 2018; Ershov et al., 2019; Lamb et al., 2019; Ngcobo et al., 2023; Fang et al., 2024; Lin et al., 2024). Among these, more than 41,000 CYP sequences have been accurately defined according to established nomenclature guidelines (Nelson, 2018). In plants, CYP enzymes have undergone extensive evolutionary diversification, driven by adaptive pressures to synthesize specialized metabolites for defense and environmental adaptation. Based on evolutionary relationships, plant CYP gene families are categorized into single-family (CYP51, CYP74, CYP97, CYP710, CYP711, CYP727, and CYP746) and multi-family (CYP71, CYP72, CYP85, and CYP86) clans (Nelson and Werck-Reichhart, 2011). Additionally, functional versatility is further amplified by gene duplication and subfunctionalization, enabling lineage-specific innovations such as alkaloid biosynthesis in *Catharanthus roseus* (Miettinen et al., 2014). Mechanistically, CYPs work via a conserved catalytic cycle involving oxygen activation and substrate hydroxylation, a process tightly coupled with cytochrome P450 reductase for electron transfer (Isin and Guengerich, 2007). The essential function of this enzyme family in primary and specialized metabolism underscores its biological significance.

CYPs play multifaceted roles in plant secondary metabolism, orchestrating the biosynthesis and modification of diverse bioactive compounds across species. Research has found that CYP-mediated metabolic pathways of endogenous hormones, including synthesis and degradation, function throughout the entire life cycle of plants. For example, *CYP76AH1* hydroxylates ferruginol to generate a cardioprotective bioactive diterpenoid tanshinone in *Salvia miltiorrhiza* (Isin and Guengerich, 2007), and *CYP716A* subfamily enzymes, such as *CYP716A53v2*, drive the oxidation of triterpenoid precursors to generate bioactive saponins in *Panax ginseng* (Han et al., 2012). While cytochrome P450 TOT1 catalyzes the oxidative rearrangement involved in the formation of the oxetane ring of paclitaxel in *Taxus mairei* (Jiang et al., 2024), the *CYP72* and *CYP93* families further drive flavonoid and triterpene diversification, whereas *CYP93G2* synthesizes momilactones, allelopathic agents that suppress weed growth in rice (Shimura et al., 2007). *CYP71B15* catalyzes the conversion of indole-3-acetaldoxime to camalexin, a phytoalexin critical for fungal resistance in *A. thaliana* (Mizutani and Ohta, 2010). Recent research has revealed that *CYP79A2* plays a role in the synthesis of dhurrin, a cyanogenic glycoside defense compound, in *Sorghum bicolor* (Pandey et al., 2019), and simultaneously, CYPs often involve metabolon formation, for example, *CYP98A3* cooperates with hydroxycinnamoyl transferases (HCTs) in lignin biosynthesis, catalyzing the 3′-hydroxylation of p-coumaroyl shikimate to caffeoyl shikimate, a critical step in monolignol production (Schoch et al., 2001; Franke et al., 2002). CYPs also orchestrate phytohormone metabolism and signaling. Multiple research studies have confirmed that CYPs influence ethylene (ET) responses through secondary metabolite synthesis or cross-talk with other hormonal pathways, and *CYP71A12* indirectly regulates ET-dependent root architecture by modulating camalexin biosynthesis in *A. thaliana* (Millet et al., 2010). Additionally, the *CYP707A* subfamily regulates abscisic acid (ABA) catabolism via 8′-hydroxylation, modulating drought tolerance (Saito et al., 2004), while *CYP94B3* hydroxylates JA-Ile to 12-OH-JA-Ile, dampening JA responses to prioritize growth under low-stress conditions in *A. thaliana* (Koo et al., 2011; Heitz et al., 2012). In tomato, *CYP94B18* and *CYP94B19* also catalyzed the oxidative catabolism of several JA–amino acid conjugates (JA–AAs), JA–Leu and JA–Val (Saito et al., 2024). These underscore their evolutionary adaptation to regulate plant stress responses (Schuler and Werck-Reichhart, 2003). Furthermore, synthetic biology employs CYPs to facilitate the microbial production of high-value compounds, exemplified by artemisinin (using *CYP71AV1* in yeast) (Paddon et al., 2013) and taxadiene (via *CYP725A4*) (Zhang et al., 2023b). These studies have highlighted the critical roles of CYPs. However, systematic identification and analysis of the CYP family members in *Hevea brasiliensis* have not yet been performed.

*H. brasiliensis* is a crucial tropical economic plant and the predominant source of natural rubber, accounting for over 40% of the total global rubber market (Cornish, 2017). Natural rubber serves as an essential raw material for manufacturing more than 50,000 distinct rubber-based products and is indispensable across industrial, medical, and military sectors. Natural rubber was synthesized within laticifer cells and constitutes a complex emulsion enriched with polyisoprene (*cis*-1,4-polyisoprene), proteins, and secondary metabolites. Recent studies have demonstrated that natural rubber biosynthesis primarily occurs through the mevalonate (MVA) and methylerythritol phosphate (MEP) pathways. Some key enzymes such as HMGR (3-hydroxy-3-methylglutaryl-CoA reductase) and CPT (*cis*-prenyltransferase) regulate the flux of isoprenoid precursors toward rubber biosynthesis (Ko et al., 2003; Long et al., 2021). Therefore, the CYP gene family, which possesses diverse biocatalytic functions, may play a role in the MVA and MEP pathways for rubber biosynthesis. Recent advancements in sequencing technologies have enabled the elucidation of the chromosome-level genome of *H. brasiliensis*, significantly expanding the research landscape in this area (Cheng et al., 2023). Despite significant progress, research on the mechanisms regulating rubber biosynthesis and stress tolerance remains limited. In recent years, an increasing number of CYP family genes have been identified in plants such as sweet potato, tomato, and eggplant (Lin et al., 2024; Tang et al., 2024; Yang et al., 2024). However, reports on the *CYP* gene family in *H. brasiliensis* are scarcely reported.

In this study, we systematically identified the *HbCYP* family genes based on *H. brasiliensis* genome sequence. Using BLASTP and Pfam domain analysis with *A. thaliana AtCYP* family genes as queries, 238 *HbCYP* genes were identified in the *H. brasiliensis* genome. Comprehensive analyses were performed, including phylogenetic tree construction, chromosomal localization, gene structure characterization, conserved motif identification, intra- and interspecies chromosomal collinearity, and cis-acting element prediction. Additionally, we examined the expression profiles of *HbCYP* genes across different tissues and their responses to methyl jasmonate (meJA) and ET treatments to explore their roles in meJA and ET signaling pathways. Differentially expressed genes (DEGs) were analyzed through Gene Ontology (GO) enrichment and validated by real-time quantitative polymerase chain reaction (RT-qPCR). Protein–protein interaction (PPI) predictions were conducted between *HbCYP* candidates and key enzymes in the rubber biosynthesis pathway. This study presents the first genome-wide analysis of the CYP superfamily in *H. brasiliensis*, with a focus on its expression under JA and ET hormonal regulation. The findings provide novel insights into the intricate regulatory mechanisms underlying rubber biosynthesis.

## 2 Materials and methods

### 2.1 Plant material

The study plants Reyan73397 were grown at the Experimental Station of the Chinese Academy of Tropical Agricultural Sciences in Danzhou, Hainan, China (19°28′ N, 109°29′ E). To confirm the expression patterns of the *HbCYP* gene family based on RNA sequencing (RNA-seq) data in response to meJA and ET treatments, latex samples were obtained from 7-year-old virgin trees subjected to a 2-g emulsion consisting of 1% ET (Sigma-Aldrich, Taufkirchen, Germany) diluted with palm oil and 1% meJA (Hao and Wu, 2000; Dai et al., 2016), employing the tapping technique as previously described (Yang et al., 2022a). Latex samples of *H. brasiliensis* were collected at 0, 4, 12, and 24 h (ET treatment) or 0, 4, 24, and 48 h (meJA treatment). The collected samples were immediately frozen in liquid nitrogen and stored at −80 °C for subsequent RNA extraction. Three biological replicates were obtained for each time point.

### 2.2 Original data and sequence collection

The chromosome-level assembly of the *H. brasiliensis* genome and the corresponding genome annotation GFF3 file were provided by the Chinese Academy of Tropical Agricultural Sciences, located in Haikou, Hainan Province, China (longitude 110°33′ E, latitude 19°98′ N). In this study, two strategies were employed to identify candidate members of the *H. brasiliensis CYP* family. First, proteins containing the CYP domain (PF00067, http://pfam-legacy.xfam.org/) were searched against the genome protein sequences using the hidden Markov model (HMM) with an *E*-value cutoff of 0.01. Second, *A. thaliana* AtCYP protein sequences (downloaded from the Arabidopsis Cytochrome P450s) were used as query sequences to search against the *H. brasiliensis* CYP protein database using the local BLASTP algorithm with *E*-values <1e^−10^. Subsequently, the candidate **HbCYP*s* were further validated using three online databases: SMART (http://smart.embl-heidelberg.de/), the NCBI Conserved Domain Database tool (https://www.ncbi.nlm.nih.gov/Structure/cdd/wrpsb.cgi), and InterPro (https://www.ebi.ac.uk/interpro/). Additionally, the physicochemical properties of the *HbCYP* family members, including amino acid (AA) size, molecular weight (MW, kDa), isoelectric point (pI), and grand average of hydropathicity (GRAVY), were analyzed using the ExPASy ProtParam tool (https://www.expasy.org/), and subcellular localization was predicted using DeepLoc 2.1 (https://services.healthtech.dtu.dk/services/DeepLoc-2.1/).

### 2.3 Phylogenetic analysis of the CYP family

In the phylogenetic tree constructed in this study, we first performed multiple sequence alignment of the full-length protein sequences of *H. brasiliensis* and *A. thaliana* using MUSCLE, the resulting alignment file was then trimmed using trim AI, and a maximum likelihood (ML) phylogenetic tree was constructed using IQ-TREE with a bootstrap value of 5,000. The classification names of *A. thaliana* AtCYPs were obtained from the *A. thaliana* Cytochrome P450s site. Based on the *A. thaliana* AtCYP classification and the CYP nomenclature rules (Nelson, 2009), we named the *HbCYP* family members. The same method was used to construct phylogenetic trees for the *H. brasiliensis* CYP family, including A-type and non-A-type CYPs. The phylogenetic trees were visualized using the iTOL platform (https://itol.embl.de/help.cgi).

### 2.4 Chromosomal distribution, conserved motif analysis, and gene structure

The chromosomal distribution of *HbCYP* genes was analyzed using TBtools-II (v2.310) (Chen et al., 2020). The gene distribution visualize tool from the GTF/GFF v3.0 module was used to display the chromosomal locations and density of *HbCYP* genes. For conserved motif analysis, the MEME online tool (Version 5.5.7) was employed with the following parameters: the number of motifs was set to 10, and the site distribution was set to “Zero or One Occurrence Per Sequence (zoops)”. The exon–intron structure and conserved motifs were visualized using the “Gene Structure View (Advanced)” tool of TBtools-II (Chen et al., 2020).

### 2.5 Genomic synteny analysis of *HbCYP* genes

The synteny analysis of *H. brasiliensis CYP* genes was performed using TBtools-II with default parameters via the “One Step MCScanX” function. The synteny data were visualized using the “Advanced Circos” tool. The genome and annotation files of *Taraxacum kok-saghyz* were downloaded from the NCBI database (https://www.ncbi.nlm.nih.gov). The whole genome sequence data of *Eucommia ulmoides* reported in this study have been deposited in the Genome Warehouse at the National Genomics Data Center, Beijing Institute of Genomics, Chinese Academy of Sciences/China National Center for Bioinformation, under accession number GWHBISF00000000 and are publicly accessible at https://ngdc.cncb.ac.cn/gwh/. The genome and annotation files of *A. thaliana* and *Morchella esculenta* were downloaded from Ensembl Plants. The synteny relationships between *H. brasiliensis CYP* genes and *T. kok-saghyz*, *M. esculenta*, *A. thaliana*, and *E. ulmoides* were analyzed using TBtools-II with default parameters via the “One Step MCScanX” function and visualized (Chen et al., 2020).

### 2.6 Gene ontology enrichment analysis

GO enrichment analysis was conducted to classify genes based on their biological processes, molecular functions, and cellular components. The latest release of the GO-basic.obo file was downloaded from the following link: http://purl.obolibrary.org/obo/go/go-basic.obo. Annotation of *H. brasiliensis* proteins was performed using eggNOG-mapper (http://eggnog-mapper.embl.de/submit_job).

### 2.7 Expression analysis of *HbCYP* genes in various tissues and treatments

RNA-seq data for analyzing the expression patterns of *HbCYP* genes across eight tissues (root, bark, leaf, latex, female flower, male flower, seed, and secondary laticifer) were obtained from the NCBI SRA database (accession numbers SRX1554800, SRX1554797, SRX1554799, SRX1554786, SRX1554813, SRX1554817, and SRX1554814) (Zhu et al., 2025). Additionally, RNA-seq data for meJA and ET treatments were obtained from the NCBI SRA under accession numbers SRP259535 and SRP504190. Transcripts per million (TPM) values and raw counts of RNA-seq data were calculated using the Kallisto Super Wrapper plugin in TBtools (default parameters). The TPM values were log2-transformed for visualization of *HbCYP* gene expression patterns. DEGs under meJA and ET treatments were identified using the Differential Gene Expression Analysis-DESeq2 Wrapper plugin in TBtools (default parameters), with screening thresholds set at |log2FC| ≥ 1 and *p*_adjust_ ≤ 0.05. Additionally, raw counts from dataset SRP259535 were utilized for Weighted Gene Co-Expression Network Analysis (WGCNA). Heatmaps visualizing tissue- and treatment-specific expression profiles were generated using TBtools-II (Chen et al., 2020).

### 2.8 Prediction of *HbCYP* PPI networks

The representative protein sequences and PPI network data of the reference species *A. thaliana* were retrieved from the STRING database (version 11.5; https://string-db.org) (Szklarczyk et al., 2022). These datasets served as a template to predict interaction relationships among *HbCYP* proteins in *H. brasiliensis*. For prediction, the “PPI Predict” module in TBtools-II was employed with a stringent minimum confidence score of 900. The PPI network was visualized and refined using Cytoscape (v3.10.0) (Otasek et al., 2019).

### 2.9 Real-time quantitative PCR validation of *HbCYP* genes

Total RNA was extracted from *H. brasiliensis* tissues subjected to ET and meJA treatments using the RNA prep Pure Plant Kit (Tiangen Biotech Co., Ltd.). Then, RNA was quantified using a NanoDrop 2000 spectrophotometer (Thermo Fisher Scientific, USA). For reverse transcription, 0.5 μg of total RNA was converted to first-strand cDNA using FastKing cDNA First Strand Synthesis Kit with genomic DNA removal (Tiangen Biotech Co., Ltd.). qPCR was performed on a LightCycler 480 II real-time PCR system (Roche, Basel, Switzerland) using the Pro Taq HS SYBR Green Premix qPCR Kit (Aikerui Biotech Co., Ltd.). Each reaction (20 μL total volume) contained 10 μL of SYBR Green Master Mix, 1 μL of cDNA template, and 0.5 μM forward/reverse primers (**Supplementary Table S8**). The actin *YLS8* gene was used as an internal reference for normalization (Li et al., 2011). Relative gene expression levels were calculated using the 2^−ΔΔCt^ method, with three biological and three technical replicates.

### 2.10 Weighted gene co-expression network analysis

Gene expression counts from 21 RNA-seq samples (the same samples used for analyzing the meJA treatment of *HbCYP*) were calculated using the Kallisto Super Wrapper plugin (default parameters) in TBtools. Subsequently, the RNA-seq counts were subjected to WGCNA. After quality assessment, 6 samples were excluded, retaining 15 samples (**Supplementary Figure 2**) for the WGCNA. The correlation analysis was performed using R as previously described (Langfelder and Horvath, 2008). An *R*² cutoff of 0.8 and a soft threshold power of 10 were selected for subsequent analyses.

### 2.11 Statistical analysis

Statistical significance was defined as *p* < 0.05, and analyses were performed using one-way analysis of variance (ANOVA) and Tukey’s multiple comparison test. Data visualization was performed using GraphPad Prism (v 9.0.0).

## 3 Results

### 3.1 Identification and characterization of *HbCYP* genes

**Figure 1:**
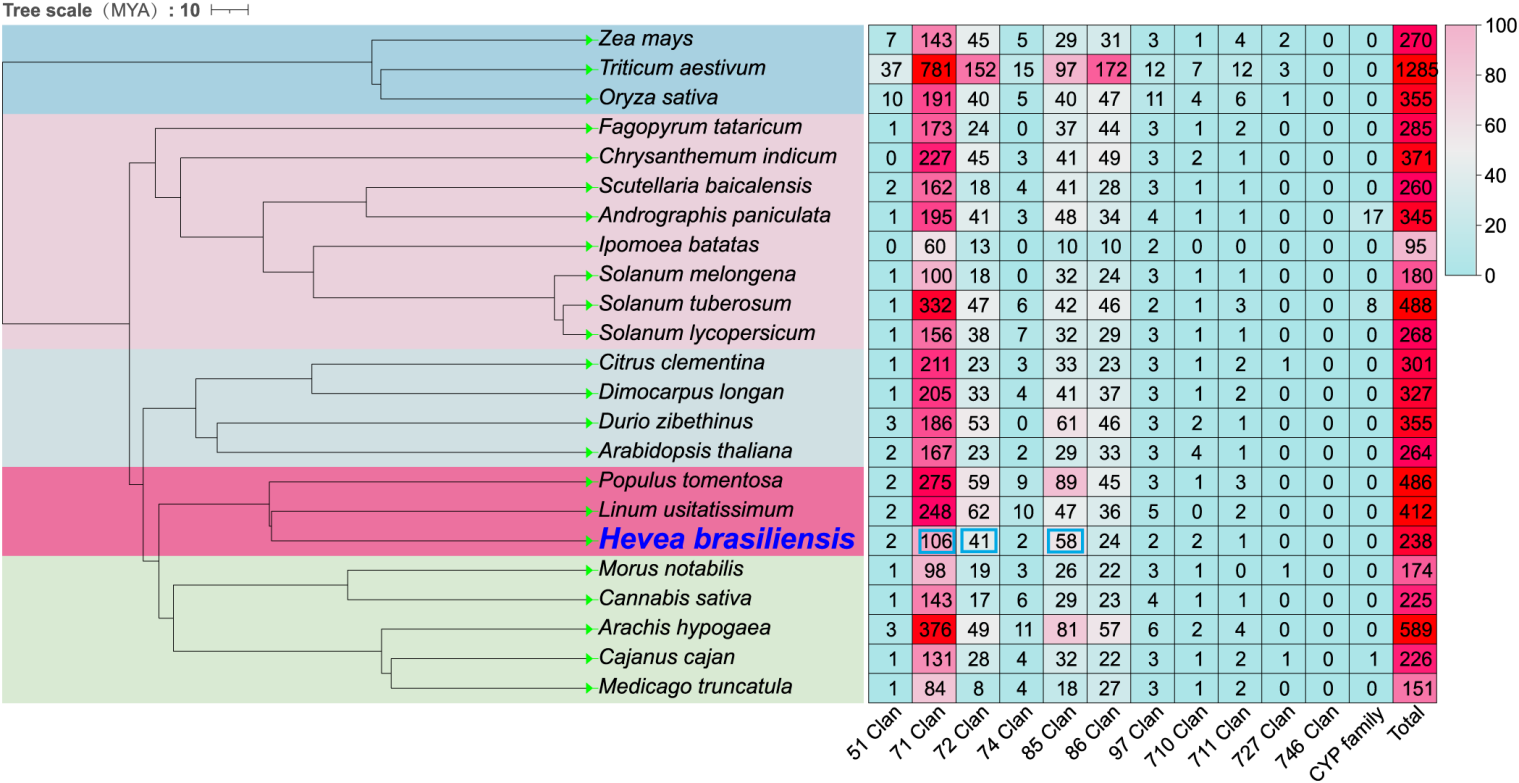
Phylogenetic analysis of H. brasiliensis and distribution of CYP Clans. Time-calibrated phylogenetic tree of H. brasiliensis and 22 additional plant species with statistical analysis of cytochrome CYP clan's member distribution. Tree scale 10, MYA (Million Years Ago). The heatmap on the right, representing CYP clan member distributions, uses a color gradient from blue to red to indicate increasing quantities.

The HMM was employed to scan for the CYP domain. Subsequently, local BLASTP searches were conducted using *A. thaliana CYP* genes as a reference. A total of 238 *HbCYP* genes were identified in *H. brasiliensis* (**Figure 1**; **Supplementary Table S1**). Additionally, the physicochemical properties of the 238 *HbCYP* proteins were analyzed using ExPASy (**Supplementary Table S1**). The results revealed that the AA lengths of these proteins ranged from 137 to 786 residues, with MW spanning 15.79 to 89.85 kDa. Other physicochemical properties, including pI and GRAVY, were analyzed (**Supplementary Table S1**). These findings revealed solubility variations among *HbCYP* family proteins; only 19 out of 238 *HbCYP* proteins showed a GRAVY value ≥0, which may influence their subcellular localization and interactions with hydrophobic substrates. Furthermore, subcellular localization prediction revealed that *HbCYP*51G1 was localized to both plastids and endoplasmic reticulum (ER), while *HbCYP*82C12 was predicted to reside in the cell membrane. Among these proteins, three *HbCYP* members were localized to the cytoplasm and nucleus, four were exclusively nuclear, six targeted plastids, and 19 were cytoplasmic. The remaining 204 proteins were all predicted to localize to the ER (**Supplementary Table S1**).

### 3.2 Classification and phylogenetic analysis of *HbCYP* proteins

**Figure 2:**
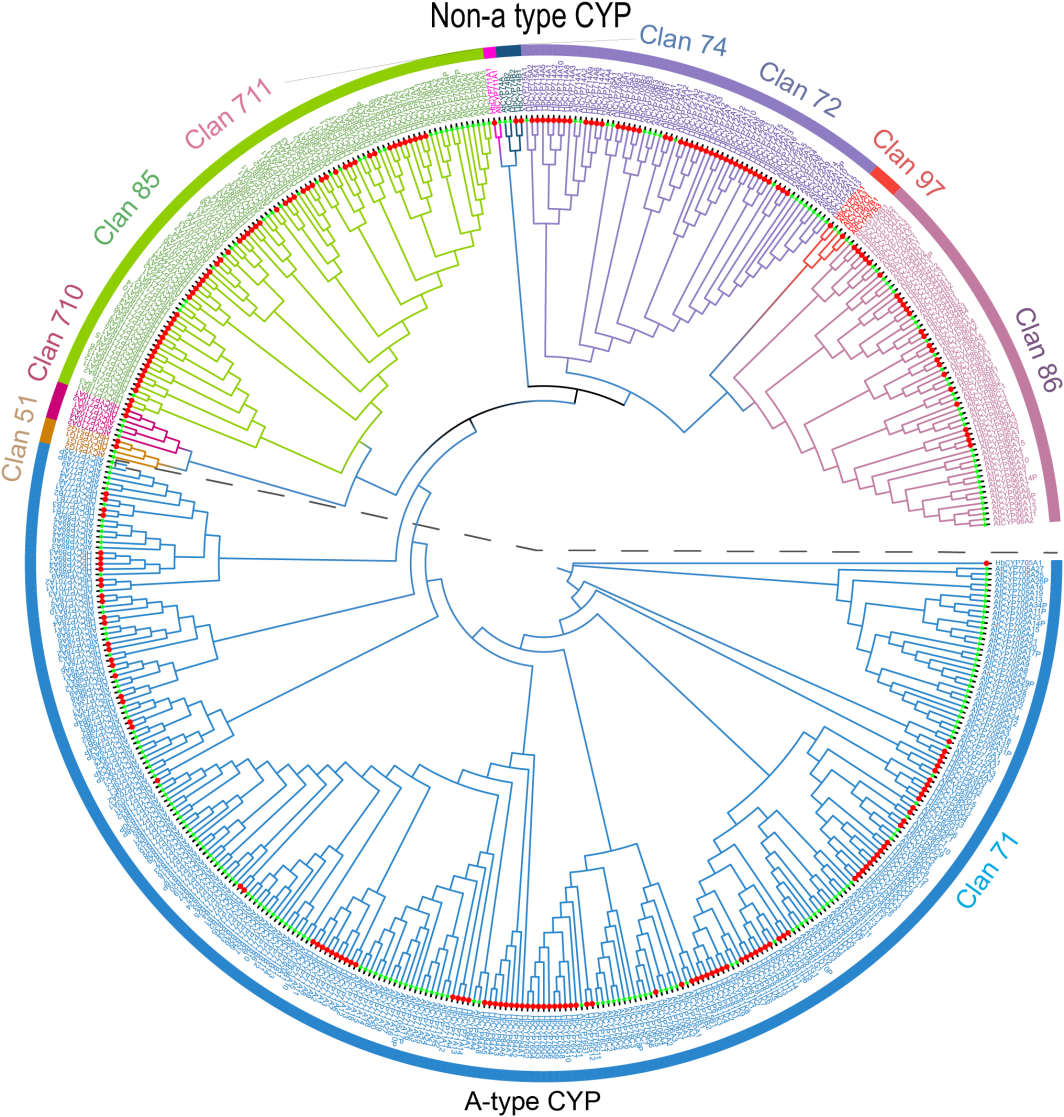
Phylogenetic analysis of CYPs in A. thaliana (272) and H. brasiliensis (238) was conducted. Phylogenetic tree was constructed using an ML method in IQTREE with best-fit model with a bootstrap value set to 5,000. Nine clans were indicated using color strips; red and green symbols indicated CYP proteins of H. brasiliensis and A. thaliana, respectively. The upper section of the dashed line indicates non-A-type CYP proteins, while the lower section designates A-type CYP proteins. The names of clans are shown outside of the circle.

To elucidate the evolutionary relationships of *H. brasiliensis*, we conducted phylogenetic analysis of 23 plant species and quantified CYP gene family clan distributions (Li et al., 2007; Ma et al., 2014; Wei and Chen, 2018; Vasav and Barvkar, 2019; Li and Wei, 2020; Sun et al., 2020, 2024; Suntichaikamolkul et al., 2021; Liu et al., 2022; Yang et al., 2024, 2024; Zhang et al., 2022, 2023a; Pei et al., 2023; Kaur et al., 2024; Lin et al., 2024, 2024; Wang et al., 2024; Chen et al., 2025; Wu et al., 2025; Yang et al., 2022b). The time-calibrated phylogeny revealed that *H. brasiliensis* shares a recent common ancestor with *Linum usitatissimum* and *P. tomentosa*. However, *H. brasiliensis* exhibits significant divergence in CYP clan composition. Its Clan 71 comprises only 106 (44.54%) members, whereas Clan 71 constitutes >52% of total CYPs in other 22 compared species, peaking at 70.10% in *Citrus clementina*. Conversely, *H. brasiliensis* shows marked expansions in Clan 72 (41 members, 17.23%) and Clan 85 (58 members, 24.37%); they are the highest proportions observed among all analyzed species (**Figure 2**), suggesting that *HbCYP* diversification may be associated with latex biosynthesis in this tropical rubber-producing plant.

To clarify the phylogenetic relationships within the *HbCYP* family in *H. brasiliensis*, an ML phylogenetic tree was constructed using 238 *H. brasiliensis* CYP members and 272 A*. thaliana* CYP members (**Figure 1**). Furthermore, the ML tree, built with the 238 *HbCYP* members, strongly supported the classification based on the P450 nomenclature (Nelson, 2009) (**Supplementary Figure S1**), confirming the reliability of the phylogenetic topology. As shown in **Figure 1**, the ML trees CYP sequences clustered into two distinct clades, A-type (designated as Clan 71) and non-A-type (including Clans 86, 85, 711, 97, 72, 710, 74, and 51). The A-type clade was subdivided into 20 families, while the non-A-type clade comprised 28 families distributed across 8 clans. These results demonstrate that the *HbCYP* family exhibits conserved evolutionary relationships with *A. thaliana* AtCYP lineages. Meanwhile, in **Supplementary Figure S1**, the A-type *HbCYP* was classified into 17 families, encompassing 106 *HbCYP* genes, whereas the non-A-type clade comprised 26 families distributed among eight clans: Clan 86 (24 **HbCYP*s*), Clan 85 (58 **HbCYP*s*), Clan 711 (1 *HbCYP*), Clan 97 (2 **HbCYP*s*), Clan 72 (41 **HbCYP*s*), Clan 710 (2 **HbCYP*s*), Clan 74 (2 **HbCYP*s*), and Clan 51 (2 **HbCYP*s*). In all *HbCYP* families, the CYP82 family (19 **HbCYP*s*) represents the largest lineage, followed by the CYP71 family (16 **HbCYP*s*). These findings provide foundational insights into the functional characterization of CYP gene families in *H. brasiliensis*.

### 3.3 Chromosome distribution, motif analysis, and gene structure of *HbCYPs*

**Figure 3:**
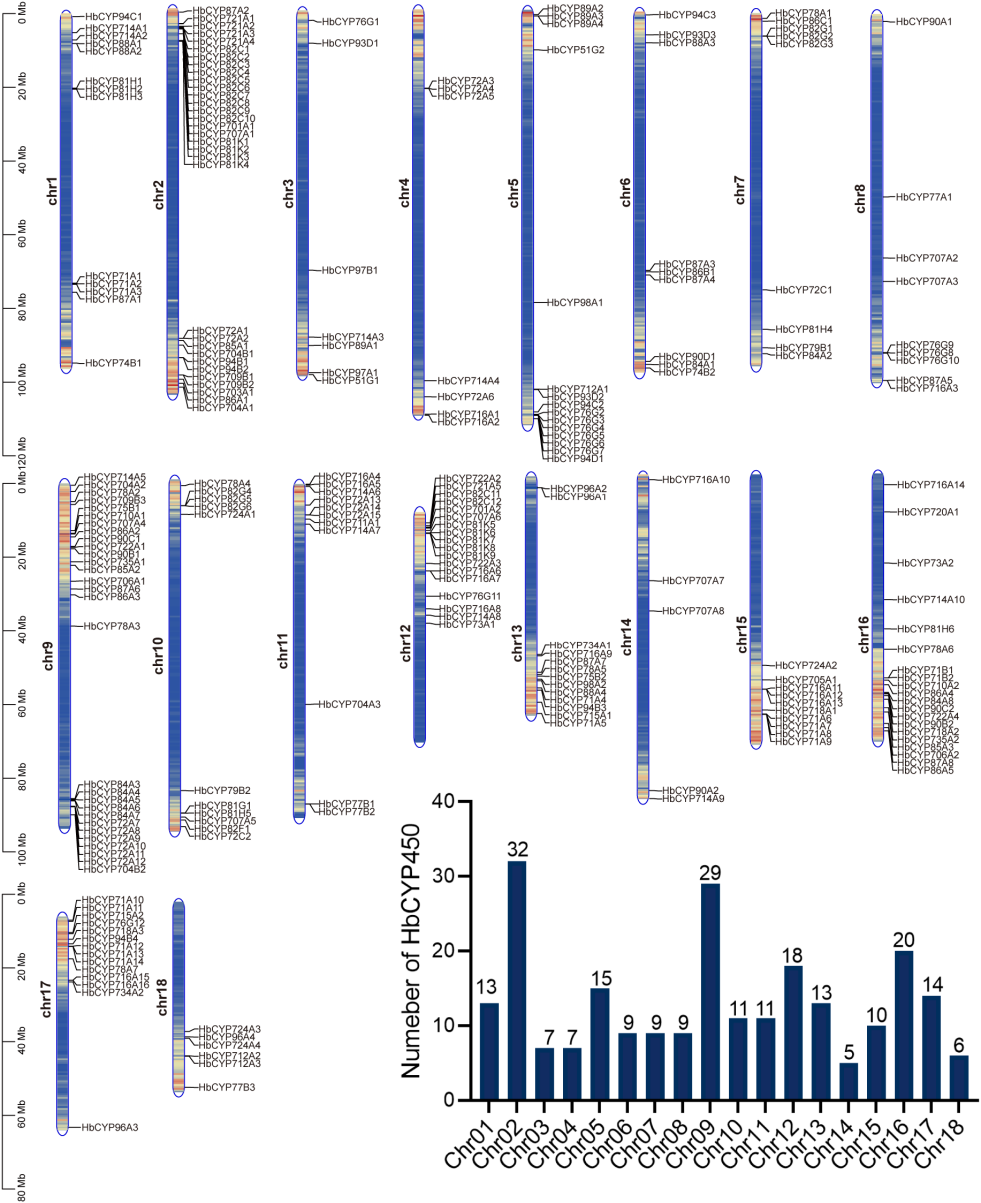
Chromosome locations of *HbCYP* genes. Chromosomal localization of *HbCYP* genes in the *H. brasiliensis* genome. Chromosome numbers (1–18) are shown at the left center of each chromosome. The color gradient from blue to orange represents increasing gene density. Black lines indicate genomic positions of individual *HbCYP* genes. The scale is in megabases (Mb). The bar chart quantifies *HbCYP* gene counts per chromosome.

**Figure 4:**
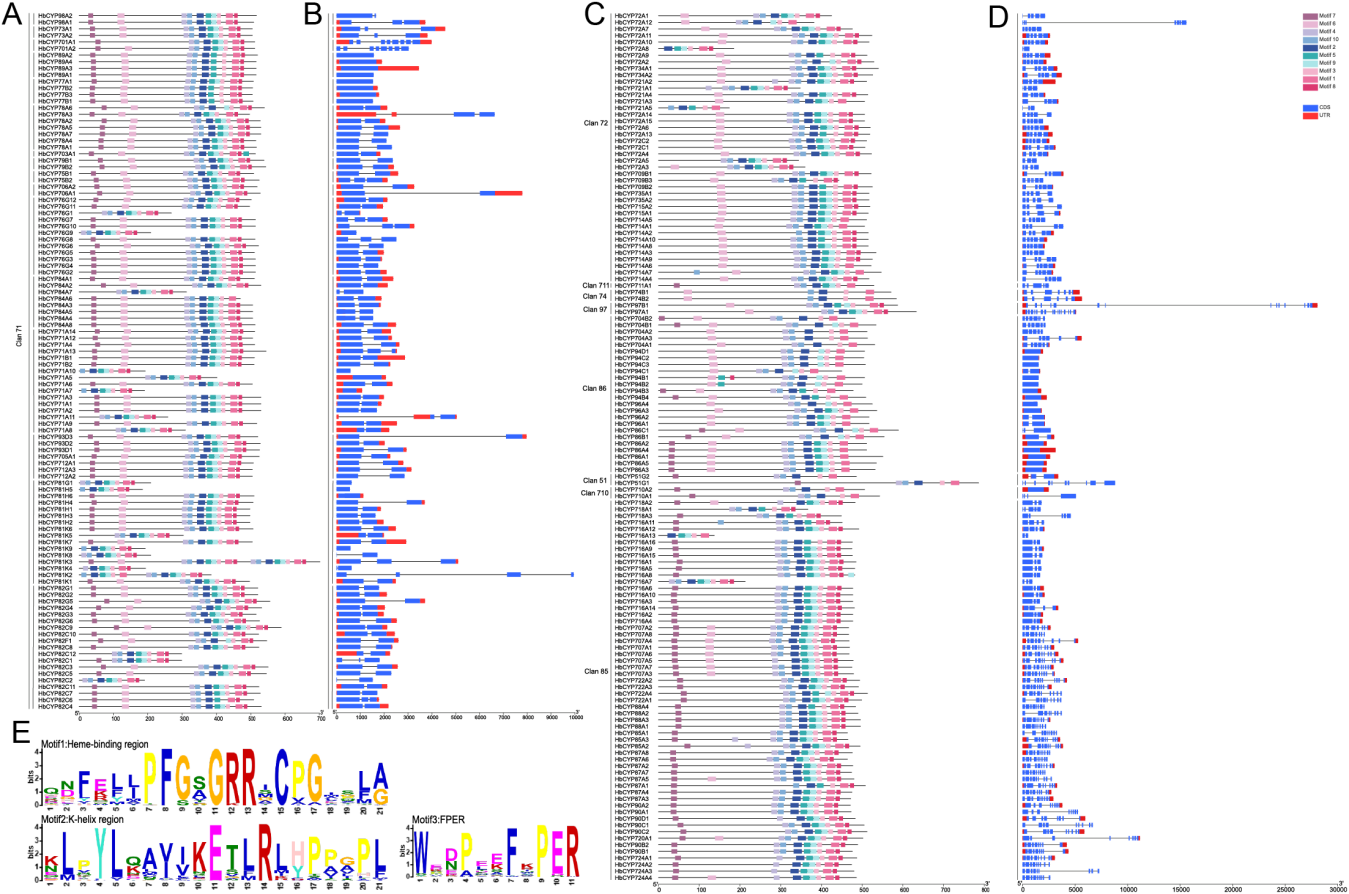
Conserved motif and gene structure of the *HbCYP* family in *H. brasiliensis.***(A, C)** Distribution of conserved motifs in A-type and non-A-type *HbCYP* proteins. Colored boxes represent motifs 1–10, with distinct colors assigned to each motif. **(B, D)** The genetic structure of the A-type and non-A-type *HbCYP* genes, including introns (black line), exons (blue rectangle), and untranslated regions (UTRs, red rectangle). **(E)** Sequence logos of three representative conserved motifs in *HbCYP* proteins (heme-binding region, K-helix region, and FPER).

Based on genomic information, the chromosomal localization of *HbCYP* genes was analyzed (**Figure 3**), the 238 *HbCYP* genes were unevenly distributed across all 18 chromosomes, with significant variation in gene numbers per chromosome. Among these, chromosome 14 harbored the fewest *HbCYP* genes (5 members), followed by chromosome 18 (6 members), while chromosome 2 contained the largest number (32 members), followed by chromosome 9 (29 members). Homologous subfamilies within gene families often share conserved motif structures, indicative of functional conservation. In this study, the AA sequences of *HbCYP* proteins were subjected to the MEME suite, identifying 10 conserved motifs (**Figures 4A, C**). Motif distribution patterns aligned with phylogenetic classification: sequences within the same clan exhibited similar motif compositions, while distinct clans showed marked divergence. For example, A-type proteins uniformly contained all 10 motifs, whereas non-A-type lineages displayed clan-specific variations. Clan 72 lacked motif 7, and within Clan 85, 58 genes predominantly retained motif 7. The strong correlation among motif architecture and phylogenetic classification underscores the robustness of the proposed subfamily delineation.

To investigate the evolutionary features of the *HbCYP* gene family, the gene structures of 238 *HbCYP* members were analyzed (**Figures 4B, D**). Distinct differences in intron–exon architecture were observed between A-type and non-A-type clades. Specifically, A-type genes exhibited highly conserved intron–exon structures, except for **HbCYP*701* (seven introns); the remaining 104 A-type **HbCYP*s* contained zero to two introns. In contrast, non-A-type genes displayed greater structural diversity; while 15 genes from Clans 86 and 711 lacked introns, the majority with more than two introns were distributed across other clans (**Figures 4B, D**). These results suggest divergent functional constraints and evolutionary trajectories between A-type and non-A-type lineage.

### 3.4 Duplication events and evolutionary characterization of **HbCYP*s*

**Figure 5:**
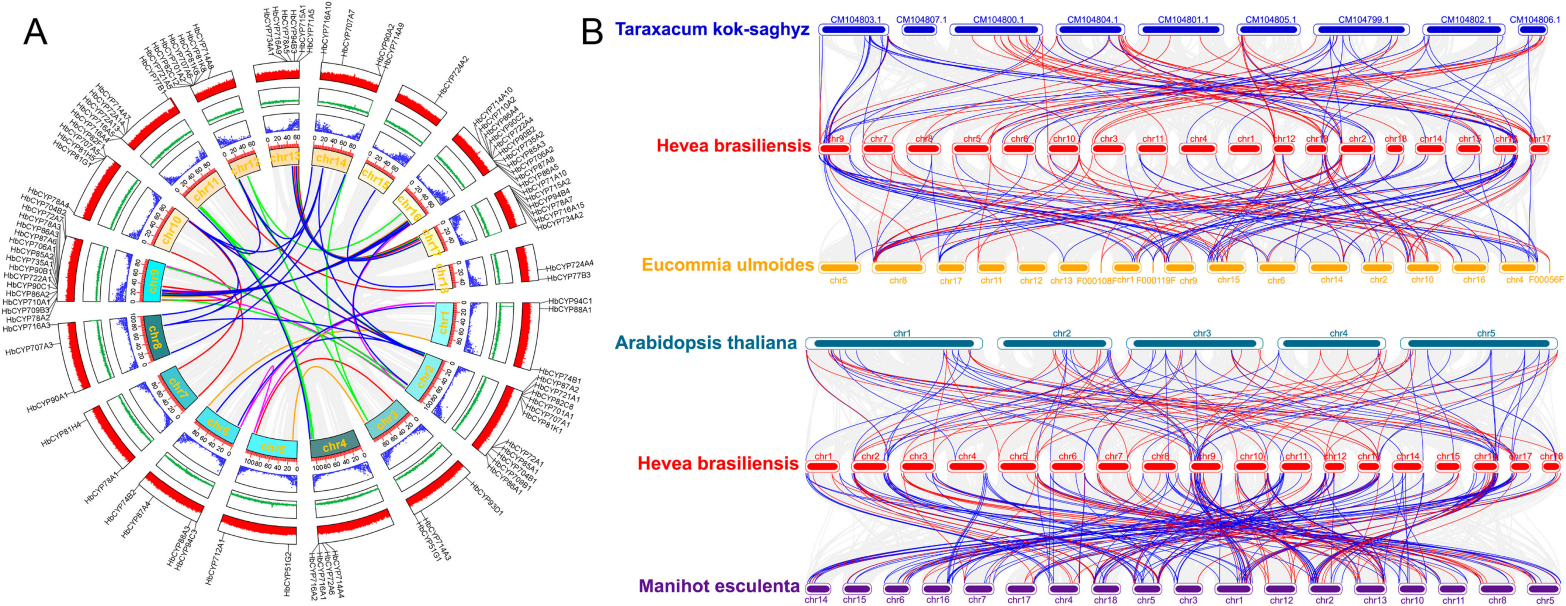
Collinearity analysis of the *HbCYP* family. **(A)** Gene duplication analysis of CYP family genes in *H. brasiliensis.* Clan 71, Clan 72, Clan 85, and Clan 86 are represented by red, blue, green, and purple lines, respectively, while other clans are depicted in orange line. The three concentric circles, arranged from the outermost to the innermost, correspond to GC content, GC skew, and gene density. **(B)** Collinearity orthologous relations among *T. kok-saghyz*, *E. ulmoides*, *A. thaliana, M. esculenta*, and *H. brasiliensis.* Clan 71 and other clans are presented by red and blue lines, respectively.

Tandem duplication events play critical roles in genome evolution, regulation, and stability. To investigate the duplication events of **HbCYP*s*, the MCScanX algorithm was employed to analyze gene duplication and synteny in the rubber tree genome (**Supplementary Table S2**). Notably, 38 (16.0%), 17 (7.1%), and 64 (26.9%) *HbCYP* genes were derived from dispersed, proximal, and tandem duplications, respectively; among these results, WGD/segmental duplications (whole-genome duplication/segmental) accounted for the largest proportion (119 *HbCYP* genes), indicating that WGD/segmental events played a predominant role in the expansion of the *HbCYP* gene family. The expansion of the *HbCYP* gene family is primarily attributed to tandem duplication and segmental duplication. Therefore, tandem and segmental duplication events were investigated using pairwise syntenic blocks generated by the MCScanX algorithm. The results revealed that 64 *HbCYP* gene pairs distributed across 18 chromosomes, with the fewest (1 pair) on chr16 and the largest number (19 pairs) on chr9, which may contribute to the large number of *HbCYP* genes (29 member*s*) on this chromosome (**Figure 5A**). These duplication events occurred across seven clans, namely, Clan 71 (14 pairs), Clan 72 (13 pairs), Clan 86 (10 pairs), Clan 74 (1 pair), Clan 85 (24 pairs), Clan 51 (1 pair), and Clan 710 (1 pair) (**Supplementary Table S2**). Furthermore, the Ka/Ks ratio was used to assess selective pressures acting on protein-coding genes in the *HbCYP* gene family of *H. brasiliensis*. The analysis revealed that only one *HbCYP* gene showed Ka/Ks > 1, indicating strong positive selection. All remaining *HbCYP* gene pairs demonstrated Ka/Ks < 0.5, suggesting predominant purifying selection (**Supplementary Table S3**). These *HbCYP* gene pairs are physically clustered on chromosomes (**Figure 3**) and form distinct clusters in the phylogenetic tree (**Figure 1**); hence, synteny analysis suggests that these genes may share functional similarities.

To clarify the evolutionary divergence of the *HbCYP* gene family in *H. brasiliensis*, comparative analyses with *T. kok-saghyz*, *E. ulmoides*, *A. thaliana*, and *M. esculenta* were performed (**Figure 5B**). The results identified 102, 139, 106, and 263 syntenic gene pairs between *H. brasiliensis* and *T. kok-saghyz*, *E. ulmoides*, *A. thaliana*, and *M. esculenta*, respectively, involving 77, 102, 72, and 148 *CYP* genes (**Figure 5B**; **Supplementary Table S4**). Further characterization revealed distinct patterns of orthologous gene retention: 54, 70, 48, and 59 *HbCYP* genes in *H. brasiliensis* possessed one ortholog in *T. kok-saghyz*, *E. ulmoides*, *A. thaliana*, and *M. esculenta*, respectively; 21, 27, 15, and 66 *HbCYP* genes harbored two orthologs across the four species; 2, 5, 8, and 20 *HbCYP* genes exhibited three orthologs in these species; and only 1 and 3 *HbCYP* genes retained four orthologs in *A. thaliana* and *M. esculenta*, respectively. These findings indicate that *HbCYP* genes in *H. brasiliensis* share closer evolutionary relationships with *E. ulmoides* and *M. esculenta* compared to *A. thaliana* and *T. kok-saghyz*. Additionally, the apparent gene loss events during speciation suggest dynamic evolutionary trajectories, providing critical insights for functional studies of *HbCYP* genes.

### 3.5 Cis-acting element analysis of *HbCYP* promoters in *H. brasiliensis*

**Figure 6:**
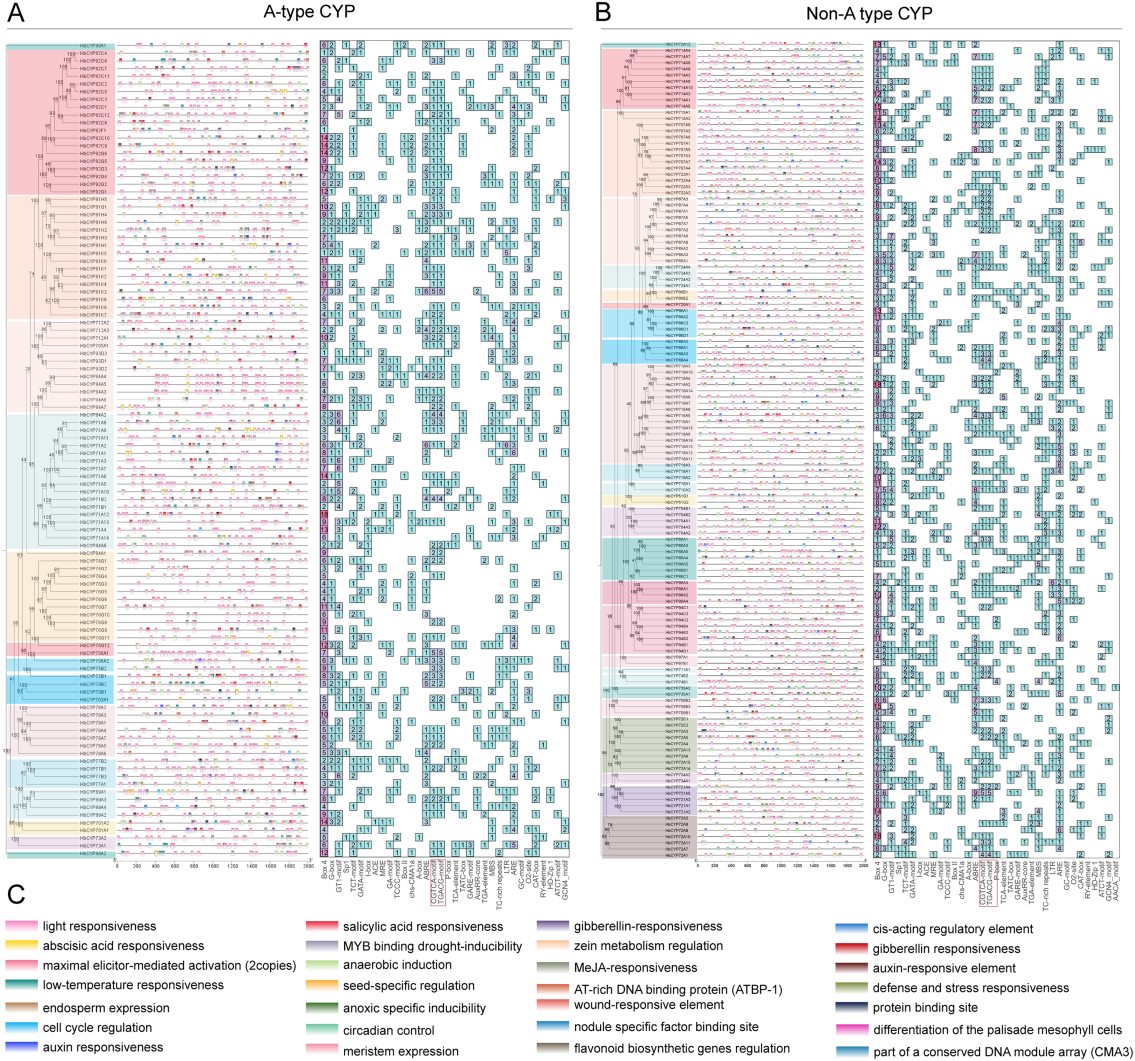
Phylogenetic tree and cis-acting element distribution. **(A)** Composition of cis-acting elements in promoter regions of A-type *HbCYP* genes. **(B)** Composition of cis-acting elements in promoter regions of non-A-type *HbCYP* genes. The black lines represent each promoter region of *HbCYP*s within 2,000 base pairs, and the differently colored squares on the lines represent various cis-acting elements. The red box on the *X*-axis indicates meJA-responsive elements. **(C)** A total of 28 key functional cis-acting elements were identified and categorized based on their roles in A-type and non-A-type *HbCYP* genes.

To investigate the transcriptional regulation of *HbCYP* genes in *H. brasiliensis*, putative promoter regions (2,000 bp upstream sequences) of 238 *HbCYP* genes were analyzed for cis-acting elements (**Figure 6**; **Supplementary Table S5**). Both A-type and non-A-type genes harbored diverse cis-acting elements associated with plant growth, hormone responses, and abiotic stress adaptation. Light-responsive elements were most abundant, with cumulative counts of 3,302 motifs (1–24 motifs per gene), predominantly comprising Box4, G-Box, GT1, and TCT motifs. In hormone-related modules, meJA-responsive CGTCA-motif and TGACG-motif were highly enriched (232 and 224 motifs in A-type and non-A-type, respectively), followed by ABA-responsive elements (ABREs; 35 motifs in A-type and 207 in non-A-type). Among abiotic stress-related elements, anaerobic induction motifs (AREs) exhibited the highest abundance (138 motifs in A-type and 214 in non-A-type). Notably, both groups contained elements linked to diverse biological processes, suggesting their potential roles in regulating growth, stress adaptation, and rubber biosynthesis in *H. brasiliensis*.

### 3.6 Expression patterns of *HbCYP* genes in tissues and treatments

**Figure 7:**
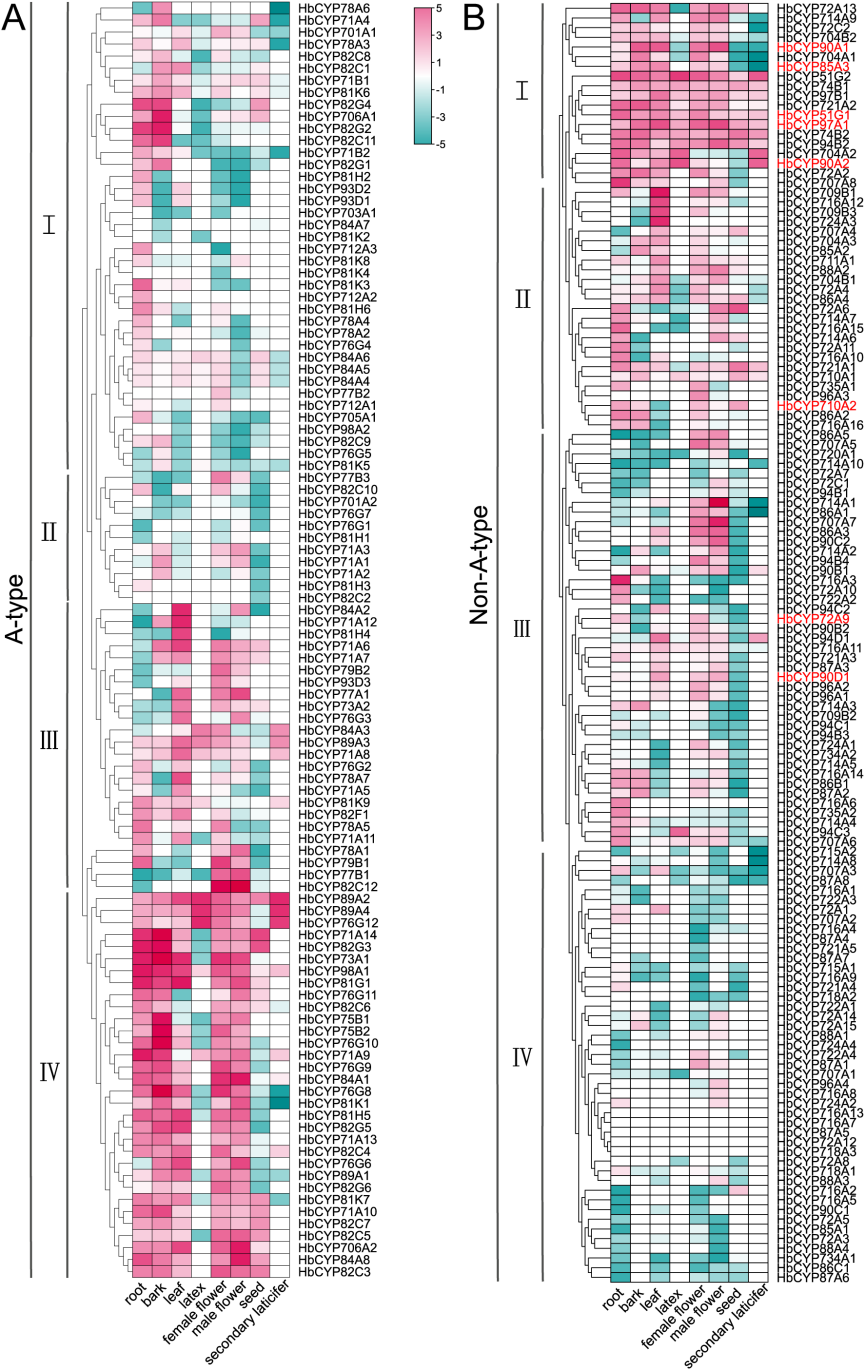
The expression patterns of **HbCYP*s* in various tissues of *H. brasiliensis*. Expression patterns of A-type **(A)** and non-A-type **(B)***HbCYP* genes were analyzed using transcriptome data from eight tissues (male flower, bark, root, seed, latex, leaf, and female flower). The color scale represents log^2^-transformed TPM-normalized counts, with blue denoting low expression and red indicating high expression. The A-type and non-A-type *HbCYP* genes were clustered into four subgroups based on expression levels. Gene names highlighted in red denote candidate genes for subsequent discussion.

**Figure 8:**
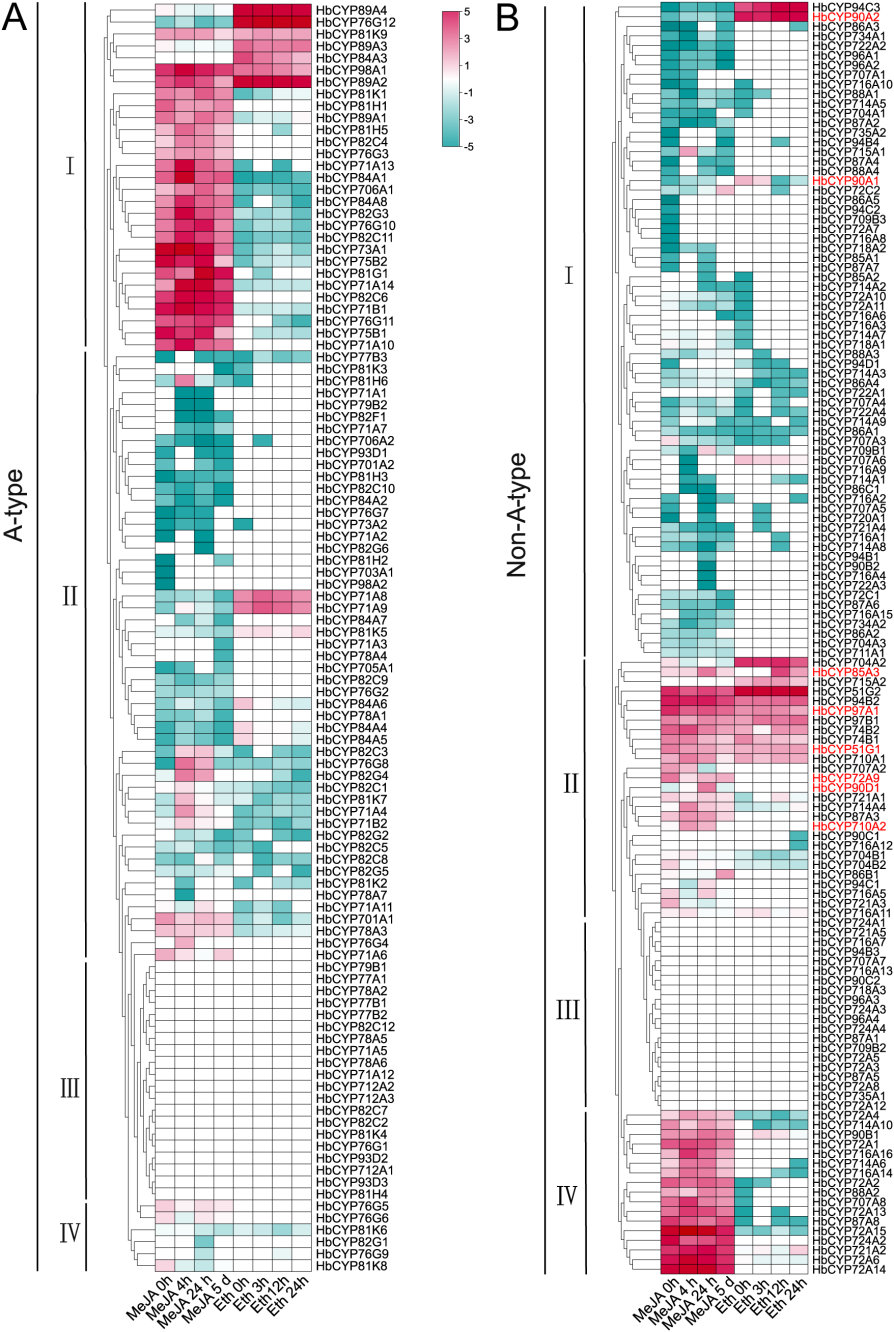
Expression profiles of *HbCYP* genes under meJA and ET treatments. Expression patterns of A-type **(A)** and non-A-type **(B)***HbCYP* genes were analyzed using transcriptome data in response to meJA (0 h,4 h, 24 h, and 5 days) and ET (0 h, 3 h, 12 h, and 24 h). The color scale represents log2-transformed TPM-normalized counts, with blue denoting low expression and red indicating high expression. The A-type and non-A-type *HbCYP* genes were clustered into four subgroups based on expression levels. Gene names highlighted in red denote candidate genes for subsequent discussion.

The tissue-specific expression profiles of *HbCYP* genes provide critical insights into their potential roles in *H. brasiliensis* development and abiotic stress responses. Eight tissues (root, bark, leaf, latex, female flower, male flower, seed, and secondary laticifer) were analyzed for the expression patterns of A-type and non-A-type *HbCYP* genes. Heatmap visualization revealed significant transcriptional divergence among the 238 *HbCYP* genes (**Figure 7**; **Supplementary Table S6**). Specifically, 10 A-type and 14 non-A-type *HbCYP* genes were highly expressed in latex and secondary laticifer. Strikingly, 55 A-type and 36 non-A-type genes were highly expressed in bark; most genes in cluster IV of A-type and cluster I of non-A-type exhibited high expression levels. Notably, **HbCYP*51G1*, **HbCYP*51G2*, **HbCYP*90A2*, **HbCYP*85A3*, and **HbCYP*97A1* exhibited pronounced highly expressed in both latex and bark, suggesting their involvement in rubber biosynthesis. Among these, **HbCYP*89A2*, **HbCYP*51G1*, **HbCYP*51G2*, **HbCYP*74B1*, **HbCYP*97B1*, **HbCYP*97A1*, **HbCYP*74B2*, and **HbCYP*94B2* were highly expressed across all tissues, suggesting the broad functional versatility of the *HbCYP* family in growth, development, and abiotic stress adaptation. Research has demonstrated that meJA and ET can influence rubber biosynthesis; therefore, we analyzed their transcriptional responses under meJA and ET treatments. Both hormonal treatments induced significant differential expression of *HbCYP* genes (**Figure 8**). Under meJA treatment, analysis (0 h, 4 h, 24 h, and 5 days) revealed dynamic expression changes. In A-type *HbCYP* genes, the number of upregulated genes was 36, 27, and 14 at 4 h, 24 h, and 5 days compared to 0 h, respectively. Conversely, 34, 23, and 22 A-type genes were downregulated at these time points. Similarly, non-A-type *HbCYP* genes exhibited 27, 14, and 9 upregulated genes and 23, 22, and 15 downregulated genes at 4 h, 24 h, and 5 days, respectively. Under ET treatment, comparisons of 0 h vs. 4 h, 12 h, and 24 h showed distinct expression patterns. For A-type *HbCYP* genes, 4, 30, and 13 genes were upregulated, while 1, 20, and 10 were downregulated at these intervals. Among non-A-type genes, 3, 25, and 11 were upregulated, with 2, 18, and 9 downregulated. In the A-type group, the majority of genes highly expressed under meJA treatment did not show elevated expression under ET treatment in cluster I. Conversely, a subset of genes displaying high expression after ET treatment were not highly expressed following meJA treatment, and an analogous expression pattern was also observed in the non-A-type group. Moreover, fewer genes exhibited similar expression patterns under both meJA and ET conditions, suggesting cross-talk between meJA and ET signaling. These results strongly suggest that the *HbCYP* gene family participates in meJA- and ET-mediated regulation of rubber biosynthesis, likely through modulating oxidative reactions and hormone-responsive pathways.

### 3.7 Gene ontology and interaction network predictions of differentially expressed *HbCYP* genes under meJA and ET treatments

**Figure 9:**
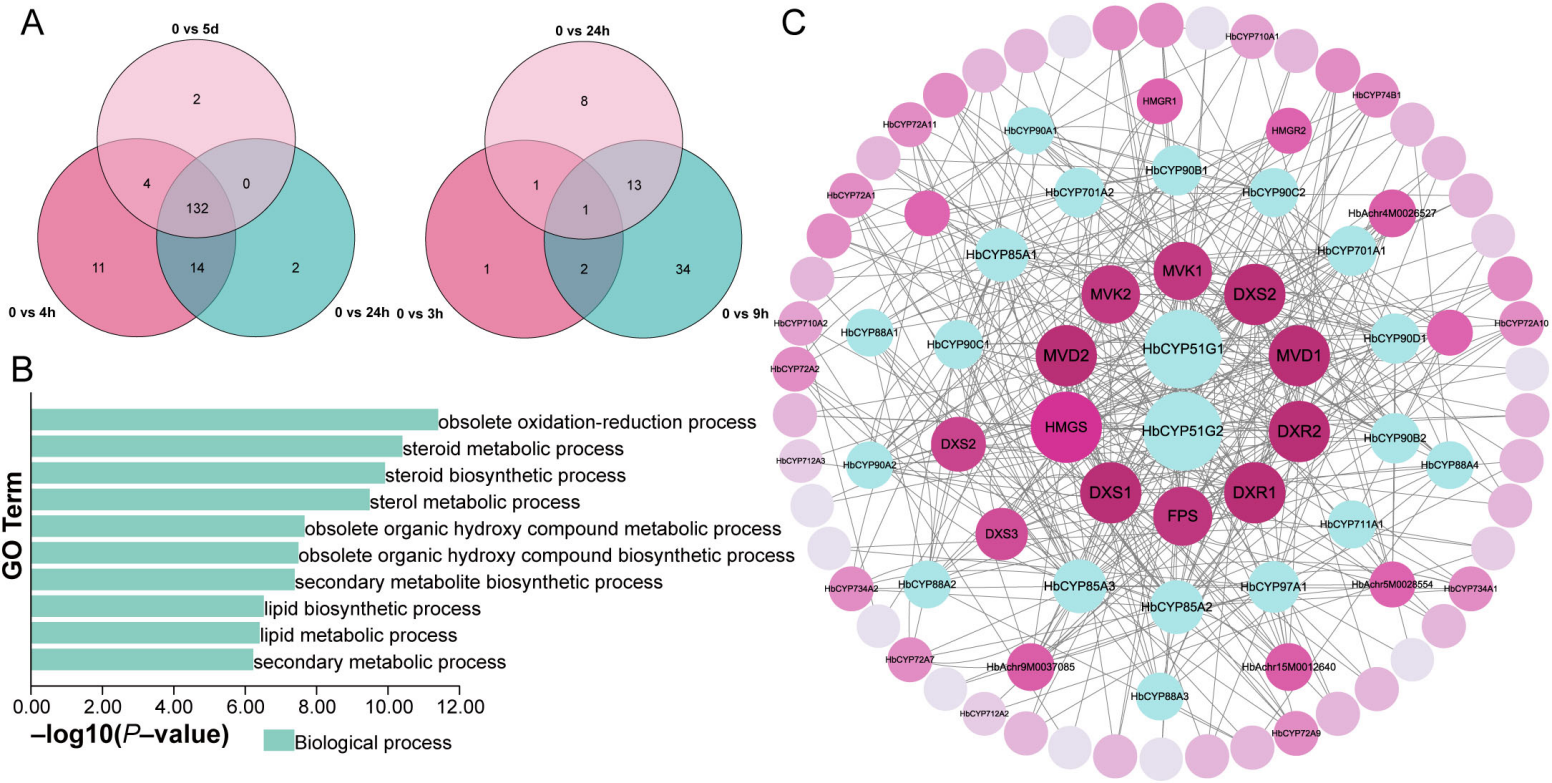
MeJA- and ET-responsive *HbCYP* genes in *H. brasiliensis*. **(A)** The number of differentially expressed *HbCYP* genes under meJA and ET treatments. **(B)** Top 10 biological process terms were displayed through GO enrichment analysis of meJA- and ET-responsive *HbCYP* differentially expressed genes. **(C)** PPI network was constructed by mapping *HbCYP* genes to *A. thaliana*. The size of the circles and their radial positioning from the center to the periphery represent the number of interaction partners. Key enzymes involved in rubber biosynthesis and identified *HbCYP* genes are labeled, while unmarked nodes represent intermediate proteins.

To investigate the roles of *HbCYP* genes in meJA- and ET-mediated rubber biosynthesis, we analyzed differentially expressed *HbCYP* genes under meJA and ET treatments (**Figure 9A**); 93 DE *HbCYP* genes were identified in meJA treatment across three comparisons (4 h, 24 h, and 5 days), including 26, 7, and 10 unique *HbCYP* genes at each time point. Notably, 14 *HbCYP* genes were expressed across all time treatment. Under ET treatment, 60 DE *HbCY*P genes were detected in comparisons of 3, 9, and 24 h, with 1, 34, and 8 unique genes at respective time points. Only 1 gene was expressed across three treatments. These findings highlight the dynamic and specific regulation of *HbCYP* genes in meJA- and ET-mediated pathways. Studies of *CYP* gene families in other plant species have demonstrated their broad involvement in diverse biological processes. To explore functional pathways, we performed GO enrichment analysis on DE *HbCYP* genes under meJA and ET treatments (**Figure 9B**). The top 10 enriched GO terms included obsolete oxidation-reduction process (GO:0055114), steroid metabolic process (GO:0008202), sterol biosynthetic process (GO:0016126), lipid biosynthetic process (GO:0008610), and secondary metabolic process (GO:0019748). These enrichments align with established roles of CYP genes in plant growth, stress adaptation, and secondary metabolism, further supporting their potential involvement in meJA- and ET-regulated rubber biosynthesis.

Natural rubber biosynthesis predominantly occurs via the MVA and MEP pathways, coordinated by multiple enzymatic steps (Tan et al., 2023). To predict *HbCYP* involvement in this process, we constructed a PPI network between *HbCYP* proteins and key enzymes in the rubber biosynthesis pathway (**Figure 9C**). The network revealed 34 **HbCYP*s* interacting directly or indirectly with core enzymes. Notably, *HbCYP*51G1 and *HbCYP*51G2 exhibited direct interactions with 3-hydroxy-3-methylglutaryl-CoA synthase (HMGS: a rate-limiting enzyme in the MVA pathway). Among these 34 **HbCYP*s*, 8 and 3 were differentially expressed under meJA and ET treatments, respectively, with **HbCYP*83A3* showing differential expression under both conditions. The PPI strongly implies that *HbCYP* genes function in rubber biosynthesis and are modulated by meJA and ET signaling, potentially bridging hormonal regulation with metabolic flux in *H. brasiliensis*.

### 3.8 Weight gene co-expression network analysis of *HbCYP*

**Figure 10:**
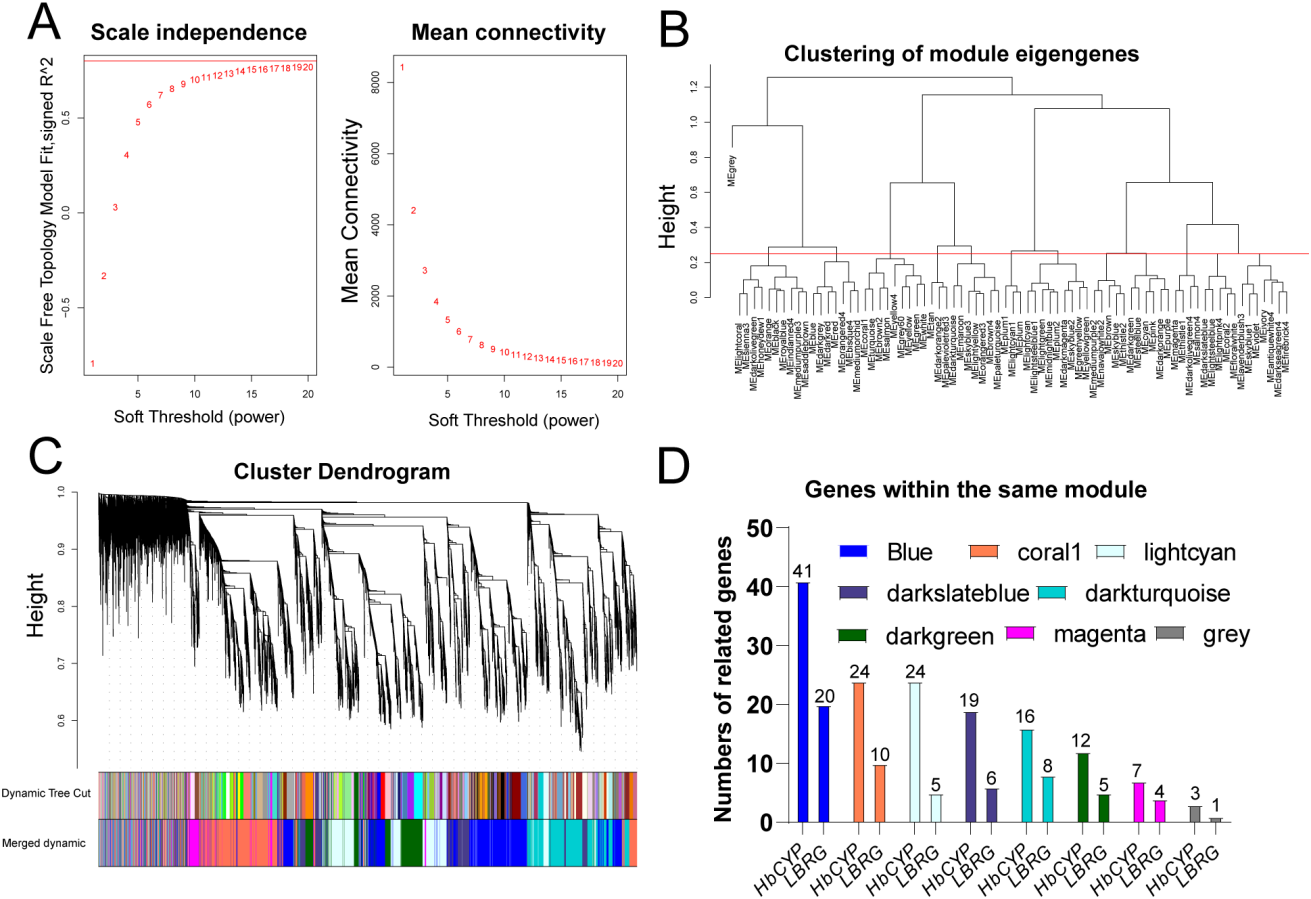
Weighted gene co-expression network analysis of *HbCYP*. **(A)** Determination of soft-thresholding power in WGCNA; the red line indicates an *R*² cutoff of 0.9. **(B)** Hierarchical clustering dendrogram with the MEgray module representing genes not assigned to any module. **(C)** Gene dendrogram with corresponding modules based on the topological overlap matrix (TOM). **(D)** Statistical analysis of *HbCYP* and LBRGs across different clustered modules, where uniform colors represent identical modules; the numerical values above the bars represent the corresponding gene counts. The color legend indicates distinct modules.

To investigate the expression relationships between the *HbCYP* gene family and latex biosynthesis-related genes (LBRGs), we employed WGCNA, an effective method for identifying co-expressed genes. Genes involved in similar biological processes or regulatory relationships are expected to cluster within the same modules. In this study, gene expression counts from 15 RNA-seq samples were subjected to WGCNA. Using a scale-free topology fitting index (*R*² = 0.8) with a soft threshold power of 10 and mean connectivity analysis (**Figure 10A**), a total of 23,286 genes passing quality filters were aggregated into color-coded modules, where the gray module represents genes not assigned to any functional module (**Figures 10B, C**). Further analysis revealed the number of co-expressed *HbCYP* genes and LBRGs clustered within the same color-designated modules (**Figure 10D**; **Supplementary Table S7**). These results demonstrate that *HbCYP* genes co-expressed with LBRGs in the same modules likely function cooperatively in rubber trees, potentially participating in natural rubber biosynthesis.

### 3.9 Potential roles of *HbCYP* genes in meJA- and ET-regulated rubber biosynthesis

**Figure 11:**
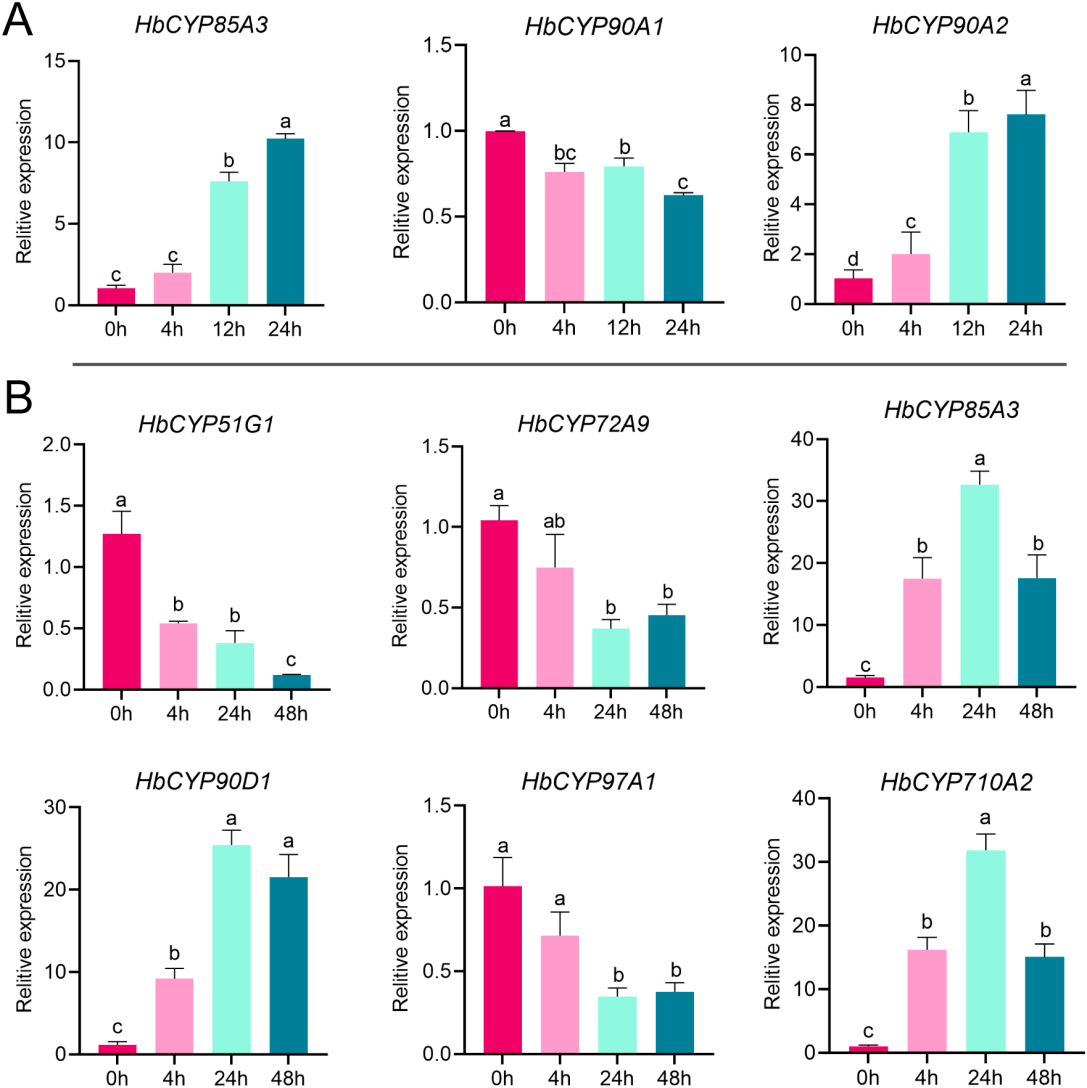
RT-qPCR analysis of representative *HbCYP* genes under ET and meJA treatments. **(A)** Expression patterns of three *HbCYP* genes under ET treatment (0, 4, 12, and 24 h). **(B)** Expression profiles of *HbCYP* genes under meJA treatment (0, 4, 24, and 48 h). One-way ANOVA and Tukey’s multiple comparison test were used to evaluate the statistical significance. Different letters indicate significance at *p* < 0.05. The error bar represents the standard deviation of three biological replicates.

To validate the expression patterns of *HbCYP* genes identified from RNA-seq data, RT-qPCR were performed on DEGs under ET and meJA treatments (**Figure 11**), with a focus on *HbCYP* genes predicted to interact with key enzymes in rubber biosynthesis. RT-qPCR results confirmed the differential expression of selected *HbCYP* genes under hormonal treatments. Specifically, genes showing upregulated or downregulated trends in RNA-seq data exhibited consistent expression tendency in RT-qPCR. For example, **HbCYP*85A3* displayed upregulation under ET (3, 12, and 24 h) and meJA (4 h, 24 h, and 5 days) treatment. Similarly, **HbCYP*90A1*, **HbCYP*51G1*, **HbCYP*72A9*, and **HbCYP*97A1* displayed downregulated trends, while **HbCYP*90A2*, **HbCYP*90D1*, and **HbCYP*710A2* displayed upregulation in RT-qPCR. The concordance between RT-qPCR and RNA-seq data indicate the reliability of our RNA-seq transcriptional profiling. These findings substantiate the critical roles of *HbCYP* genes in ET- and meJA-mediated signaling pathways, likely modulating rubber biosynthesis through interactions with relative enzymes.

## 4 Discussion

### 4.1 Members and characterization of *HbCYP* genes

*H. brasiliensis* serves as the global primary source of natural rubber, with its derivatives being indispensable for tire manufacturing, medical applications, and industrial products, constituting a critical strategic commodity in modern economies (Cornish, 2017). With the advancement of genome sequencing technologies, novel CYP genes from diverse organisms have continuously isolated, establishing a comprehensive CYP database encompassing animals, plants, fungi, bacteria, viruses, and other life forms (Sello et al., 2015; Khumalo et al., 2020; Fang et al., 2024). However, the genome-wide analysis of the *HbCYP* gene in *H. brasiliensis* remains not reported. In this study, we conducted a comprehensive identification and characterization of the *HbCYP* family in *H. brasiliensis* genome, and a total of 238 *HbCYP* genes were identified through local BLASTP, HMMER, CDD, and conserved motif analyses (**Figure 1**; **Supplementary Figure S1**). Generally, subcellular localization is closely associated with potential biological functions. In this study, the subcellular localization prediction results suggest that the *HbCYP* family may participate in the regulation of natural rubber biosynthesis through compartmentalized distribution. WGCNA further indicated that some *HbCYP* co-clustered with latex biosynthesis related genes in specific modules. Among the 238 identified *HbCYP* members, 86% (204) were localized to the ER, implying their potential involvement in terpenoid precursor synthesis or rubber chain elongation. Notably, the dual localization of *HbCYP*51G1 (plastids and ER) may mediate cross-membrane compartmental coordination between the MVA pathway and rubber biosynthesis. Additionally, the six plastid-localized members might regulate isoprenoid precursor supply, while the plasma membrane localized *HbCYP*82C12 could participate in transmembrane transport or signal transduction. The cytoplasmic (19) and nuclear (4) members may influence rubber biosynthesis pathways through metabolic flux regulation or transcriptional control. This highly specialized subcellular distribution pattern suggests that the *HbCYP* family coordinates natural rubber biosynthesis and storage in laticifers through spatial division of labor.

### 4.2 Phylogenetic tree, chromosomal localization, and gene structure of *HbCYPs*

The analysis of P450 family clans showed that Clan 71 was contracted, whereas Clans 72 and 85 were expanded in *H. brasiliensis* (**Figure 2**). These divergences likely correlate with species-specific metabolic demands, particularly rubber biosynthesis. Specifically, Clan 71 contraction may reflect a redirected metabolic focus toward rubber-related compounds (e.g., polyisoprenoid biosynthesis), whereas the expansions of Clans 72 and 85 potentially enhance enzymatic capacity for these specialized pathways. Additionally, genomic events during evolutionary selection may serve as potential drivers underlying the observed clan composition divergence. In land plants, *CYP* genes are categorized into A-type *P450s* and non-A-type *P450s* (Nelson and Werck-Reichhart, 2011). In this study, 238 *HbCYP* genes were classified into two major clades and nine clans (**Figure 1**; **Supplementary Figure S1**). Among these clans, Clan 71 contained the largest number of *HbCYP* genes, consistent with observations in most angiosperms (Wei and Chen, 2018; Li and Wei, 2020). Notably, only two **HbCYP*51G* genes were identified in *H. brasiliensis*, and as the most evolutionarily conserved eukaryotic CYPs, CYP51 enzymes exclusively mediate sterol 14α-demethylase activity across fungi, plants, and animals (O’Brien et al., 2005). Researches revealed that *CYP51* gene copy numbers range from 7 to 37 in rice, maize, and wheat, while *CYP51H* subfamily members vary from 7 (rice), 3 (maize), to 29 (wheat) (Wei and Chen, 2018; Li and Wei, 2020). Phylogenetically, CYP51, CYP710, and CYP85 clans clustered within a large monophyletic branch (**Figure 1**; **Supplementary Figure S1**). Prior studies indicate that CYP710s function downstream of CYP51s in sterol biosynthesis, whereas Clan CYP85 members are implicated in terpenoid-related pathways, BR biosynthesis, and ABA catabolism (Vriet et al., 2013). Additionally, gene duplication serves as a primary driver of functional diversification and adaptive evolution (Wendel, 2000), suggesting that this clustering pattern reflects tandem duplication events underlying *HbCYP* family expansion. Moreover, all *HbCYP* gene pairs exhibited KA/KS ratios<0.5 except for one pair with ratio >1 (**Supplementary Table S3**), indicating that the *HbCYP* gene family has undergone purifying selection. As previous research has shown, motif prediction identified three signature domains present in all *HbCYP* genes, motif 1 (heme-binding domain), motif 2 (K-helix: ExxR), and motif 3 (PERF: PxRx) (Li et al., 2007; Lamesch et al., 2012). Members of the same clan generally share similar motifs, indicative of conserved functional characteristics within each clan. Additionally, non-A-type members exhibited greater structural complexity, particularly in intron number, and this aligns with reports in rice, wheat, and maize (Wei and Chen, 2018; Li and Wei, 2020). The elevated intron count in non-A-type genes likely stems from frequent gene duplication and structural reorganization during evolution, facilitating functional plasticity for diverse secondary metabolic demands (Paquette et al., 2000; Mizutani and Ohta, 2010; Nelson and Werck-Reichhart, 2011). These structural divergences may underpin substrate-binding specificity and functional diversification among *HbCYP* genes.

### 4.3 Genome evolution, transcriptional element, and expression pattern analysis

Researches have revealed extensive gene duplication events in angiosperms such as *Citrus* spp. and wheat, driven by selective pressures to enhance environmental adaptability (Li and Wei, 2020; Liu et al., 2022). Intraspecific synteny analysis across *H. brasiliensis* chromosomes indicated that WGDs/segmental duplications primarily drove *HbCYP* family expansion while tandem duplications played a complementary role. Furthermore, interspecific synteny analysis revealed stronger conservation with *M. esculenta*, approximately double the number detected in *E. ulmoides*, *A. thaliana*, and *T. kok-saghyz* (**Figure 5B**; **Supplementary Table S4**). These results demonstrate closer phylogenetic proximity to *M. esculenta* and suggest frequent gene loss in orthologous lineages during speciation, leading to differential retention of *HbCYP* homologs across taxa. Cis-acting elements serve as pivotal molecular switches in transcriptional regulatory networks, playing indispensable roles during plant responses to diverse biotic and abiotic stress (Lescot et al., 2002; Maston et al., 2006; Wray, 2007). In *H. brasiliensis*, these elements are functionally associated with biological processes (**Figure 6**; **Supplementary Table S5**), which is consistent with previous reports demonstrating the involvement of CYP gene families in light-responsive pathways (Jensen et al., 2011). Furthermore, abundant meJA-responsive elements were identified (**Figure 6**; **Supplementary Table S5**), aligning with the established roles of CYP enzymes in meJA signaling (Schaller and Stintzi, 2009; Hansen et al., 2021; Wang et al., 2023). Functional prediction of these regulatory elements supports the involvement of *HbCYP* genes in modulating plant growth and developmental processes. Tissue-specific gene expression patterns provide insights into functional specialization. In this study, RNA-seq analysis revealed distinct expression profiles of *HbCYP* genes across tissues (**Figure 7**; **Supplementary Table S6**). Previous studies have demonstrated that rubber biosynthesis and production is regulated by meJA and ET (Johnson et al., 2021; Chao et al., 2023; Li et al., 2025). Tissue-specific expression profiling further revealed their potential functional roles; among these genes, **HbCYP*89A2* exhibited high expression across tissues, and previous studies have demonstrated *CYP89A2*’s functional role in triterpenoid saponin biosynthesis (Zhang et al., 2021), which is derived from the MVA or MEP pathway. Notably, latex and bark tissues displayed pronounced differential expression of *HbCYP* genes. Five genes (**HbCYP*51G1/2*, **HbCYP*90A2*, **HbCYP*85A3*, and **HbCYP*97A1*) were significantly upregulated in latex and bark, with their expression further modulated by meJA or ET treatments (**Figures 7**, **8**). These results suggest that certain *HbCYP* genes regulated by meJA or ET may participate in rubber biosynthesis.

### 4.4 Potential roles of *HbCYP* genes in rubber biosynthesis

In this study, PPI predictions indicated binding between *HbCYP*51G1/2 and HMGS. Given that CYP51 enzymes catalyze obtusifoliol 14α-demethylation during phytosterol biosynthesis (Jiao et al., 2020), we hypothesize that *HbCYP*51–HMGS complexes may enhance substrate channeling, analogous to HMGR–SMT1 interactions (Boutté and Grebe, 2009); this hypothesis requires further validation. Moreover, GO enrichment analysis of meJA/ET-responsive genes suggests their potential involvement in rubber biosynthesis (**Figure 9B**); PPI networks connected differentially expressed **HbCYP*s* with key rubber synthesis enzymes (**Figures 9A, C**). Furthermore, WGCNA results revealed that co-expressed *HbCYP* genes clustered with LBRGs in different modules (**Figure 10D**), suggesting functional coordination in rubber production pathways. RT-qPCR validation showed expression patterns consistent with the RNA-seq data (**Figure 11**), confirming the potential roles of these *HbCYP* genes in meJA/ET-modulated rubber biosynthesis. These results suggest that the *HbCYP* family may participate in meJA/ET-regulated pathways to modulate rubber biosynthesis.

## 5 Future research

This study provides the first systematic characterization of the *HbCYP* family in *H. brasiliensis*, highlighting their potential roles in rubber biosynthesis. However, the underlying mechanisms remain to be elucidated. Further functional validation through genetic transformation is required to confirm the biological significance of *HbCYP* genes. Further investigations are required to elucidate the molecular mechanisms of meJA/ET-regulated rubber biosynthesis, particularly the functional interactions between **HbCYP*s* and key metabolic components such as HMGS. The specific contributions of expanded clans (e.g., Clans 72 and 85) to rubber biosynthesis also remain unresolved. Future studies should prioritize the functional dissection of *HbCYP* genes via CRISPR-mediated mutagenesis to delineate their roles in rubber synthesis. Furthermore, multi-omics integration was performed to elucidate *HbCYP*-associated secondary metabolic networks and their correlations with rubber synthesis traits. These studies could unravel novel targets for molecular breeding aimed at enhancing rubber productivity.

## Data Availability

The datasets presented in this study can be found in online repositories. The names of the repository/repositories and accession number(s) can be found in the article/**Supplementary Material**.
