# Supplementary material for: Genome-wide identification and expression profile of CYP genes in rubber tree (Hevea brasiliensis)
Source: Front Plant Sci. 2026 Apr 22;17:1750005. doi: 10.3389/fpls.2026.1750005 (PMC13144162; doi:10.3389/fpls.2026.1750005)
Supplement: Supplementary file 2 [file Table2.docx]

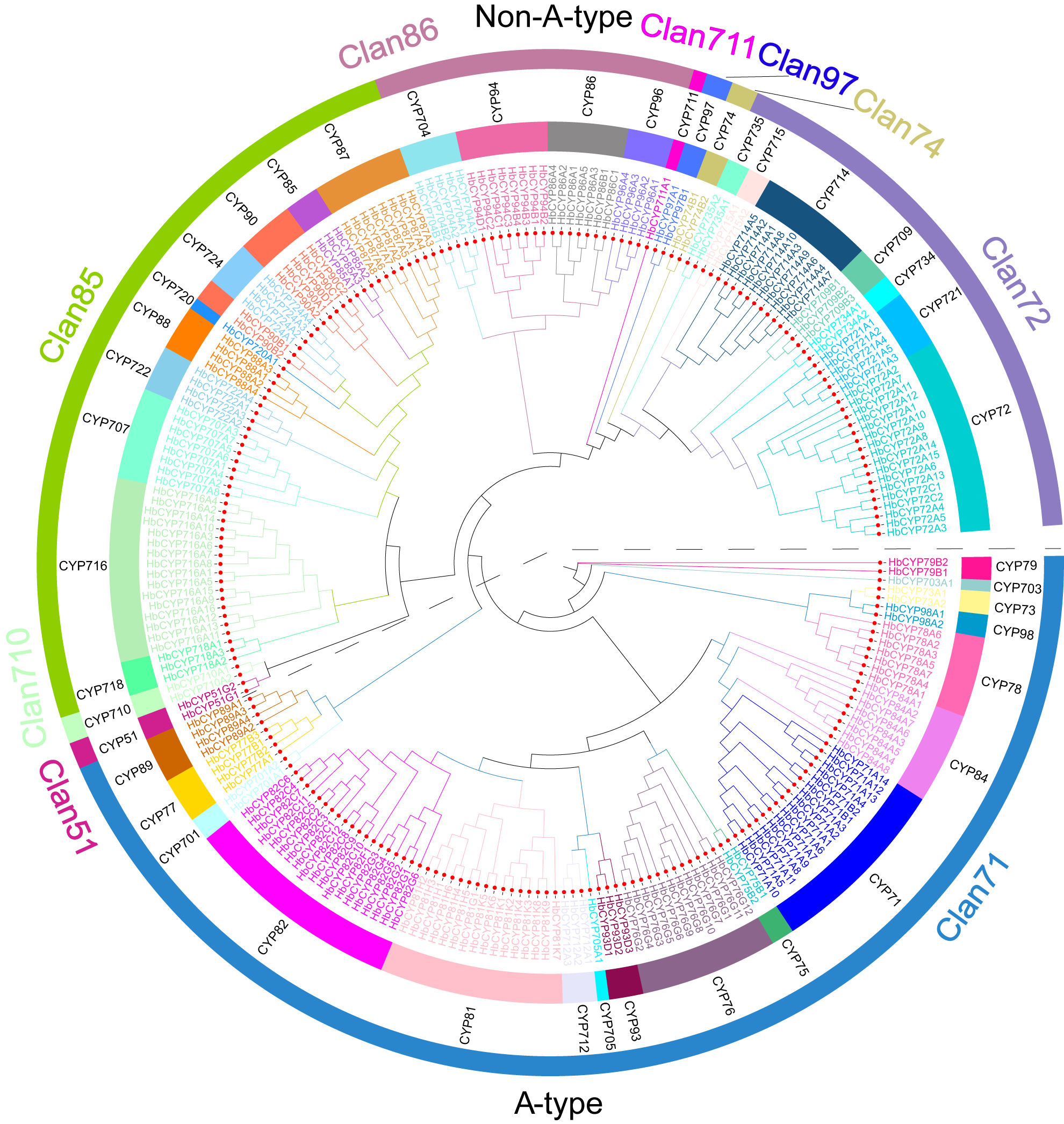


**Supplementary Figure S1. Phylogenetic analysis of CYP450s in *H. brasiliensis* was conducted.** Phylogenetic tree was constructed using a ML method in IQTREE with best fit model with a bootstrap value set to 5000. The upper section of the dashed line indicates non-A-type CYP proteins, while the lower section designates A-type CYP proteins. The outermost ring featuring color-coded strips representing 9 clans (clan names annotated outside each corresponding strip), while the inner ring illustrates 43 subfamilies through differentially colored segments, where each unique color corresponds to a specific subfamily classification.


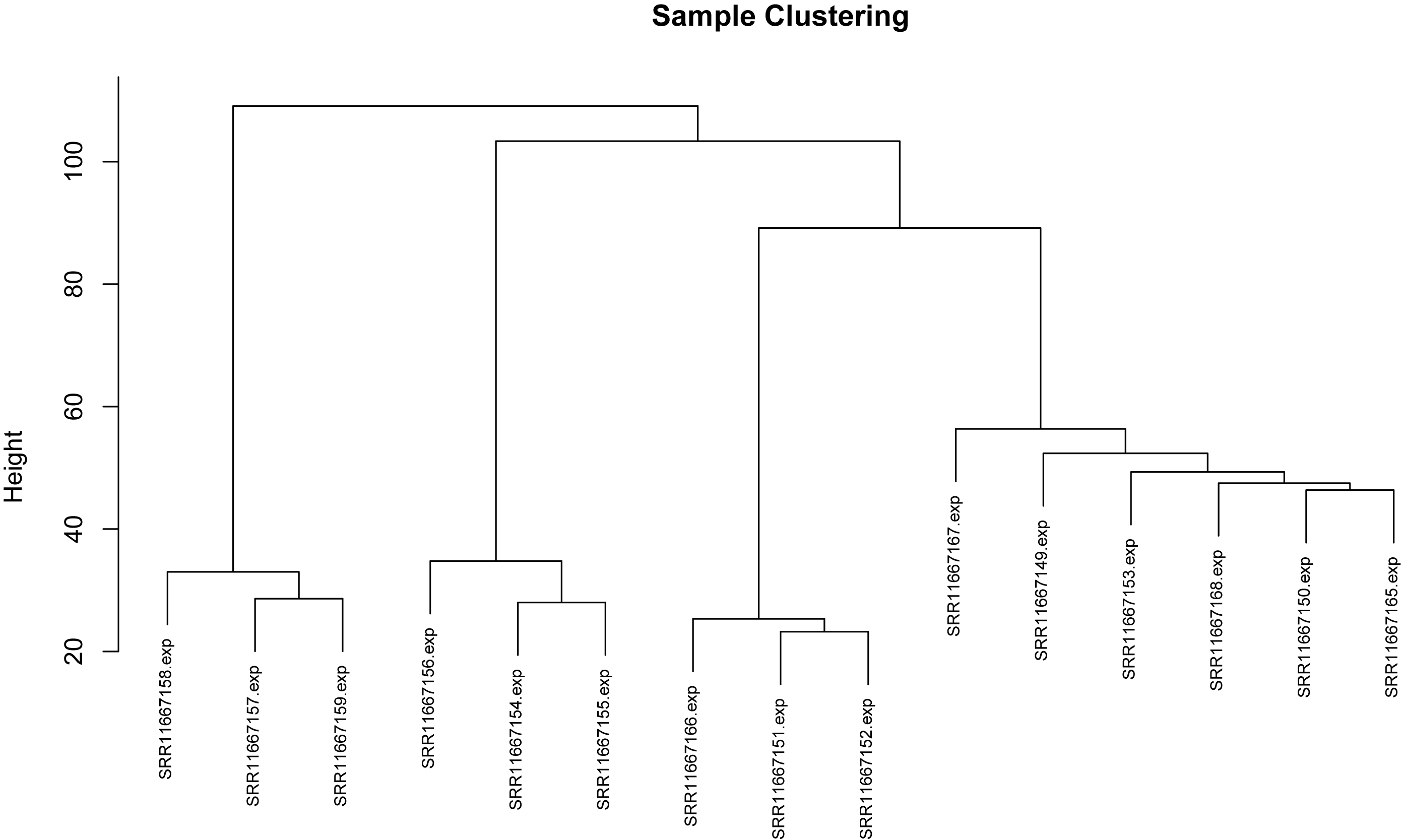


**Supplementary Figure S2.** Cluster dendrogram of the 15 samples used for WGCNA.
